# Nanomicelles for GLUT1-targeting hepatocellular carcinoma therapy based on NADPH depletion

**DOI:** 10.1080/10717544.2022.2162160

**Published:** 2022-12-29

**Authors:** Congyi Zhang, Zehui Liu, Feng Wang, Bin Zhang, Xirui Zhang, Peiwen Guo, Tianwei Li, Sheng Tai, Changmei Zhang

**Affiliations:** a Department of Hepatic Surgery, Second Affiliated Hospital of Harbin Medical University, Harbin, China; b Department of Children’s and Adolescent Health, Public Health College, Harbin Medical University, Harbin, China; c Department of Pharmaceutics, Daqing Campus of Harbin Medical University, Daqing, China

**Keywords:** Hepatocellular carcinoma, nanomicelles, GLUT1-targeting, NADPH depletion

## Abstract

Hepatocellular carcinoma (HCC) is a malignant tumor leading cancer-associated high mortality worldwide. Unfortunately, the most commonly used drug therapeutics not only lack of target ability and efficiency, but also exhibit severe systemic toxicity to normal tissues. Thus, effective and targeted nanodrug of HCC therapy is emerging as a more important issue. Here, we design and develop the novel nanomicelles, namely Mannose-polyethylene glycol 600-Nitroimidazole (Man-NIT). This micelle compound with high purity comprise two parts, which can self-assemble into nanoscale micelle. The outer shell is selected mannose as hydrophilic moiety, while the inner core is nitroimidazole as hydrophobic moiety. In the cell experiment, Man-NIT was more cellular uptake by HCCLM3 cells due to the mannose modification. Mannose as a kind of glucose transporter 1 (GLUT1) substrate, can specifically recognize and bind to over-expressed GLUT1 on carcinoma cytomembrane. The nitroimidazole moiety of Man-NIT was reduced by the over-expressed nitroreductase with reduced nicotinamide adenine dinucleotide phosphate (NADPH) as the cofactor, resulting in transient deletion of NADPH and glutathione (GSH). The increase of reactive oxygen species (ROS) in HCCLM3 cells disturbed the balance of redox, and finally caused the death of tumor cells. Additional in vivo experiment was conducted using twenty-four male BALB/c nude mice to build the tumor model. The results showed that nanomicelles were accumulated in the liver of mice. The tumor size and pathological features were obviously improved after nanomicelles treatment. It indicates that namomicelles have a tumor inhibition effect, especially Man-NIT, which may be a potential nanodrug of chemotherapeutics for HCC therapy.

## 1. Introduction

Hepatocellular carcinoma (HCC), one of the malignant diseases with high incidence rate, is the most common liver cancer that seriously threatens human health, and it is also the leading cause of death (Llovet et al., 2003). At present, early HCC can be effectively treated by radical treatment measures, such as surgical resection, liver transplantation and local ablation. However, due to the occult onset and rapid progress of this disease, most of the patients with HCC are in the middle and late stage after being diagnosed, and the overall treatment effect is not ideal (Liu et al., 2020). Chemotherapy are being used as first-line treatment methods against HCC in advanced stage, including tyrosinase inhibitors lenvatinib and sorafenib, doxorubicin, brefeldin A, etc. But the objective remission rate is 2–3% (Kudo et al., 2018; Zhang et al., 2021). This approach has demonstrated promising results in previous clinical studies, but its efficacy still varies between different chemotherapeutic agents due to lack of targeted capacity and high nonspecific toxicity (Takemura & Fujiwara, 2007; Chatterjee et al., 2010). Herein, development of a kind of highly effective and targeted nanodrugs is emerging as a promising therapy strategy for HCC.

Nowadays, nanomedicine has attracted an increasing interest in tumor therapy for the sake of improving poor solubility, bioavailability, significant toxicity and short half-time of hydrophobic antitumor drugs and prolonging blood circulation by resisting rapid renal clearance (Phillips et al., 1993; Kikuchi et al., 2003; He et al., 2013). Nanomicelles, a kind of nano-delivery systems, especially self-assembled polymer micelles, are characterized by nanosized shape with hydrophilic shell and hydrophobic core, which can form self-assembly structure in aqueous medium. Hydrophobic shell is selected as storage for carrying drugs, genes and proteins, while hydrophilic core can protect the encapsulated drugs from the external environment, maintain the spatial stability of particles, and achieve sustained and slow drug release (Zhang et al., 2017; Chai et al., 2020). In addition, the targeted ligand is modified on the polymer micelle by chemical methods, which can deliver the drug to the targeted site to achieve active targeted delivery (Bazak et al., 2015). The hydrophobic core can also be directly used as an antitumor drug. Compared with the method of being encapsulated drug by common micelles, this approach can improve the drug loading and exhibit maximal outcome of therapeutic effect.

Glucose transporter 1 (GLUT1), an essential member of the glucose transport protein family, is mainly responsible for bidirectional D-glucose transmembrane transport to maintain appropriate concentrations in cells (Wood & Trayhurn, 2003). Glucose metabolism in tumor cells is also known as Warburg effect (Lu, 2019), in which more glucose will be depleted in tumor cells to promote proliferation under hypoxia. Therefore, GLUT1 is highly expressed in tumor cells and will be a potential target for drug delivery in tumor cells (Vander Heiden, 2011; Szablewski, 2013). Mannose, the epimer of glucose, can specifically recognize and bind to over-expressed GLUT1 on tumor cell surfaces as a targeted ligand (Zhang et al., 2020). The nanodrug modified by mannose will be highly transported and taken in by GLUT1 to enhance the delivery efficiency and achieve active targeted ability.

Most tumor cells grow rapidly and proliferate continuously to induce the Warburg effect and oxidative stress, which in turn destroys the redox system of tumor cells (Szatrowski & Nathan, 1991). Excessive reactive oxygen species (ROS) produced by oxidative stress is toxic to cells. It is reported that tumor cells may be more vulnerable to ROS damage than normal cells (Ramsey & Sharpless, 2006; Trachootham et al., 2006). In fact, tumor cells can escape oxidative damage by enhancing their intrinsic antioxidant defense system, thereby reducing ROS levels. However, when the tumor cells are forced to accumulate excessive ROS, they will die due to their peroxidation. Therefore, the purpose of killing tumor cells can be achieved by regulating redox to control the level of ROS in tumor cells.

There are several important redox material pairs in cells, among which NADP^+^/NADPH and oxidized/reduced glutathione (GSSG/GSH) are more closely involved in the redox process of the body. GSH will be oxidized to GSSG, and GSSG can also be reduced to GSH with nicotinamide adenine dinucleotide phosphate (NADPH) participation, which makes the GSH/GSSG redox be in a relatively constant equilibrium state. It will lead to redox imbalance when the GSH generation is insufficient, too much NADPH is consumed, or GSSG cannot be reduced in time (Smith et al., 1996). It is believed that the action of NADPH involves the generation and contribution of GSH as the cofactor. NADPH will participate in the process of GSSG to reductive GSH (Deponte, 2013; Gorai et al., 2022). Logically, NADPH depletion will lead to decrease of GSH. Therefore, researchers design a kind of self-assemble micelles, in which the hydrophobic core is nitroimidazole. The nitroimidazole moiety is reduced by the over-expressed nitroreductase with reduced NADPH as the cofactor, resulting in transient deletion of NADPH and GSH (Guo et al., 2020). Finally, the imbalance of redox in tumor cells results in tumor cell death.

**Scheme 1.**
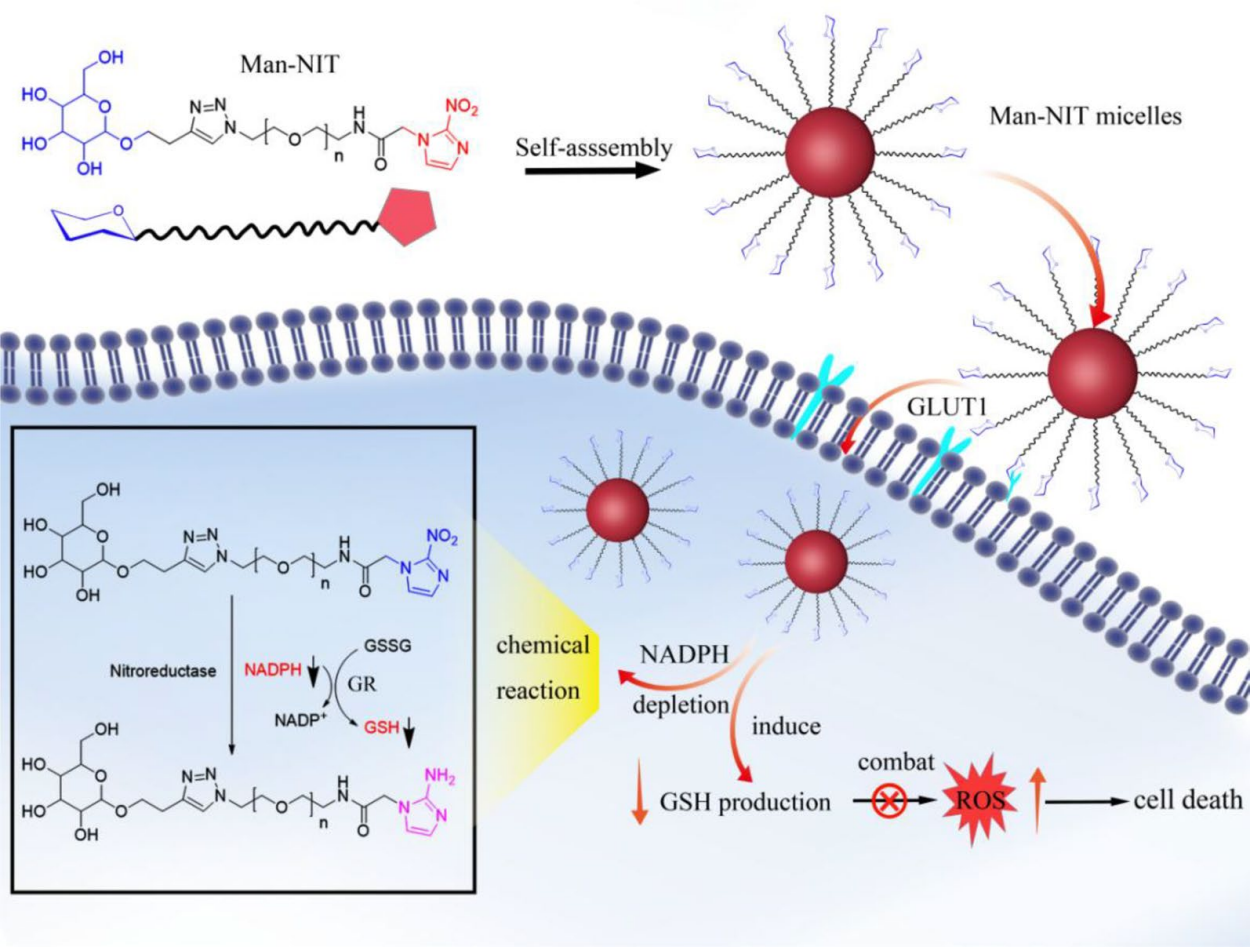
Schematic illustration of the action mechanism of Man-NIT micelles.

Based on the theory above, we develop a structurally novel targeted self-assembled nanomicelles, namely Man-NIT, which can actively target to the HCC with the assistance of GLUT1. The hydrophobic moiety of Man-NIT is nitroimidazole compound, which will deplete NADPH, directly inhibit GSH synthetize, and increase ROS, resulting in tumor growth inhibition, as shown in Scheme 1. This research realizes the targeted therapy of nanomicelles for HCC.

## 2. Materials and methods

### 2.1. Materials

Human HCC cell lines HCCLM3 cells were obtained from the Chinese Academy of Science (Shanghai, China); Dulbecco Modified Eagle Medium (DMEM), Fetal Bovine Serum (FBS) were purchased from GIBCO; Penicillin-streptomycin, Trypsin, MTT, Apoptosis Kit, Reactive Oxygen Species Assay Kit, NADP^+^/NADPH detection kit, GSH detection kit, Cell apoptosis kit, Calcein AM-PI staining kit were purchased form Beyotime Biotechnology; 3,3′-Dioctadecyloxacarbocyanine perchlorate (DiO) (SIGMA-ALDRICH), HE staining solution, 4% paraformaldehyde, Silica gel plate (Merck, Germany) and 5-(4, 6-Dichlorotriazinyl) aminofluorescein (DiR) (Macklin) were purchased from Innochem. Sephadex LH-20 was purchased from Shanghai McLean Biochemical Technology Co., Ltd.

Male BALB/c nude mice (4–6 weeks old) were purchased from Beijing Vital River Laboratory Animal Technology Co., Ltd. NO.: 220518514622. All mice were fed in SFP level animal room. All animal experiments were approved by animal ethics committee of Harbin Medical University (KY2019-046). Ki67 antibody (ab16667), second antibody goat anti-rabbit IgG (BA-1000) were purchased form Abcam.

Liquid mass spectrometer UPLC-LCT Premier XE (Walters, USA); High performance liquid chromatography LC-20T (Shimadzu, Japan); Nano-ZS90 nanoparticle size and ζ-potential analyzer (Malvern, British) ; SYNERGY microplate reader (BIOTEK, USA); Rotary evaporator (EYELA, Japan); Transmission electron microscope imaging (TEM) (JEM1200EX, Japan); Flow cytometry (CytoFLEX, Beckman); Live Cell Station (GE, USA); Fluorescence microscope (Leica, German); The ^1^H NMR spectra of the compounds were tested by Dalian University of technology.

### 2.2. Methods

#### 2.2.1. Synthesis of Man-NIT and PEG-NIT

##### 2.2.1.1. Synthesis of compound 1 and 2

**Scheme 2.**
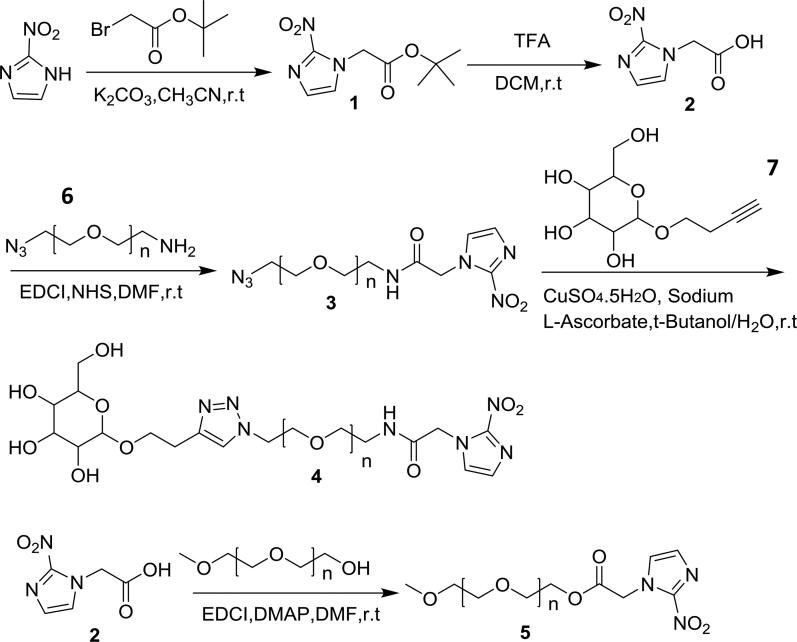
The synthetic route of Man-NIT and PEG-NIT.

For details, refer to Scheme 2 for the synthesis route of all chemical products. Nitroimidazole (256 mg, 2.2.7 mmol), potassium carbonate (1.25 g, 9.08 mmol) were dissolved in acetonitrile (30 mL) under an argon atmosphere in a round**-**bottomed flask. The mixture was stirred at room temperature for 30 min. The mixture was stirred at room temperature overnight after being added Tert-Butyl bromoacetate (530.4 mg, 2.72 mmol). The solvent of resulting crude mixture was removed by vacuum, dissolved in CH_2_Cl_2_ and washed with 1 M aqueous HCl. The organic layer was dried over sodium sulfate, filtered, and removed excess solvent under reduced pressure to afford the product **1**.

Compound **1** was dissolved in TFA/CH_2_Cl_2_ (6 mL/6 mL) under an argon atmosphere in a round-bottomed flask. The mixture was stirred at room temperature overnight, upon which time the solvent was removed by vacuum. The resulting crude solid was dissolved in ether, filtered and dried at room temperature to afford the pure product **2** (337 mg, two-step yield 87%): HRMS (ESI) Calcd for C_5_H_5_N_3_O_4_[M + H]^+^ 171; found 171.

##### 2.2.1.2. Synthesis of compound 3

Compound **1** (214 mg, 1.25 mmol), N-hydroxysuccinimide (144 mg, 1.25 mmol) and 1-(3-dimethylaminopropyl)-3-ethylcarbodiimide hydrochloride (239 mg, 1.25 mmol) were dissolved in DMF (10 mL) under an argon atmosphere in a round**-**bottomed flask. The mixture was stirred at room temperature for 30 min. Compound **6** (500 mg, 0.83 mmol) were added to the mixture and stirred at room temperature overnight. The resulting crude mixture was washed with 1 M aqueous HCl. The organic layer was dried over sodium sulfate, filtered, and removed excess solvent under reduced pressure. The crude product was purified by flash column chromatography on silica gel (MeOH/CH_2_Cl_2_ 1:10) to afford the pure product **3** (470 mg, 74.9%): HRMS (ESI) Calcd for C_(9 + 2n)_H_(13 + 4n)_N_7_O_(4+n)_ [M + H]^+^ 724.4, 768.4, 812.4 et al.; found 724.4, 768.4, 812.4 et al.

##### 2.2.1.3. Synthesis of compound 4

Compound **3** (470 mg, 0.62 mmol) was dissolved in tert-butanol (10 mL) in a round-bottomed flask. A solution of compound **7** (431 mg, 1.86 mmol) in H_2_O (10 mL), 0.05 M aqueous CuSO_4_ (380 μL, 0.19 mmol) and 0.05 M aqueous sodium ascorbate (1.24 mL, 0.62 mmol) were added. The mixture was stirred at room temperature overnight. The solvent of crude mixture was removed by vacuum, dissolved in CH_2_Cl_2_ and then filtered the insoluble impurities. The crude product was purified by flash column chromatography on silica gel (MeOH/CH_2_Cl_2_ 1:1 0 ∼ 1:5) to afford the pure product **4** (Man-NIT).

##### 2.2.1.4. Synthesis of compound 5

Compound **2** (171 mg, 1 mmol), mPEG600 (500 mg, 0.67 mmol) 1-(3-dimethylaminopropyl)-3-ethylcarbodiimide hydrochloride (191 mg, 1 mmol), 4-dimethylaminopyridine (85.4 mg, 0.7 mmol) were dissolved in DMF (10 mL) under an argon atmosphere in a round-bottomed flask. The mixture was stirred overnight. The resulting crude mixture was washed with 1 M aqueous HCl. The organic layer was dried over sodium sulfate, filtered, and removed excess solvent under reduced pressure. The crude product was purified by flash column chromatography on silica gel (MeOH/CH_2_Cl_2_ 1:10) to afford the pure product **5 (PEG-NIT)**.

#### 2.2.2. Characterization of nanomicelles

##### 2.2.2.1. Nanomicelles preparation

Man-NIT and PEG-NIT micelles were prepared at room temperature, and they were diluted to 1.0 mg/mL by deionized water. The average particle size, polydispersity index (PDI), ζ-potential were detected by Malvem Zetasizer Nano ZS 90 (Malvern, UK). TEM images of nanomicelles were obtained using a Tecnai G2 20 S-TWIN instrument. Man-NIT and PEG-NIT micelles were diluted to 0.5 mg/mL by deionized water and the ultraviolet/fluorescence absorption spectra were detected using SYNERGY microplate reader.

##### 2.2.2.2. Stability

Man-NIT and PEG-NIT micelles were stored at 4 °C for 30 days, and sampled in 0, 5, 10, 15, 20, 25, 30 days for evaluating the stability of nanomicelles by measuring the particle size and PDI. Moreover, the nanomicelles were diluted 10, 50, 100, and 500 times by deionized water, respectively. The dilution stability was evaluated by measuring the particle size and PDI of nanomicelles.

##### 2.2.2.3. Hemolytic activity

Man-NIT and PEG-NIT micelles (1.0 mg/mL) were diluted to 500, 250, 125, 62.5, 31.25 μg/mL for sample solution preparation. A red blood cell suspension (2% v/v) was obtained and added to the sample solution. The mixture was gently mixed and incubated at 37 °C for 1 h. After centrifugation at 2500 rpm for 10 min, the supernate (100 μL) was taken and added to 96-well plates. At last, the absorbance was detected at 540 nm. Distilled water and normal saline mixed with 2% red blood cell suspension were used as positive control (100%) and negative control (0%), respectively. Hemolysis ratio% = (Asample-Anegative)/(Apositive-Anegative)×100%.

#### 2.2.3. Cell experiment

##### 2.2.3.1. Cellular uptake

DiO were encapsulated with nanomicelles to prepare the Man-NIT@DiO, PEG-NIT@DiO (1 mg/mL). HCCLM3 cells were cultured in 12-well plates overnight with a concentration of 1 × 10^5^ cells/mL and continued to incubate with 20 μL Man-NIT@DiO and PEG-NIT@DiO for 4 h. Cells were washed with PBS twice and digested 3–4 min with 0.25% trypsin. The cells were centrifuged at 2000 rpm for 3 min to remove supernatant. Cell precipitation was collected and resuspended in 0.5 mL PBS. Cell suspension was measured using flow cytometry with FITC channel.

##### 2.2.3.2. MTT assay

HCCLM3 cells were seeded at a density of 1 × 10^4^ cells/mL in 96-well plates for about 24 h. Then, cells were incubated with Man-NIT and PEG-NIT micelles with different concentration for 24 h. After 24 h, 100 μL fresh culture medium and 10 μL MTT (5 mg/mL) were added for another 4 h. MTT-containing solution was removed and 100 μL of DMSO was added to dissolve the MTT formazan crystals. At last, the mixture was detected at 490 nm after vibration 10 min.

##### 2.2.3.3. NADPH content

HCCLM3 cells were cultured at a density of 1 × 10^5^ cells/mL in 6-well plates and treated with Man-NIT and PEG-NIT micelles (1.0 mg/mL) for 24 h. The content of NADPH was detected in each group according to the instructions of the NADP^+^/NADPH detection kit.

##### 2.2.3.4. GSH content

HCCLM3 cells were cultured at a density of 1 × 10^5^ cells/mL in 6-well plates and treated with Man-NIT and PEG-NIT micelles (1.0 mg/mL) for 24 h. The cells were washed with PBS, collected after centrifugation, and the supernatant was sucked up. Three times the cell precipitation volume of protein removal reagent solution was added. The samples were then subjected to two rapid freeze-thawing processes using liquid nitrogen and 37 °C water bath. The samples were placed at 4 °C for 5 min and centrifuged at 10,000 g for 10 min. The supernatant was removed and detected at 412 nm.

##### 2.2.3.5. ROS detection

HCCLM3 cells were cultured at a density of 1 × 10^4^ cells/mL in 12-well plates. The cells were treated with Man-NIT and PEG-NIT micelles (1.0 mg/mL, DMEM) for 24 h. The cells were washed with DMEM for 3 times to remove the culture medium. DCFH-DA (10 μmol/L) was diluted to 1:1000 and added to the cells. The cells were incubated with DCFH-DA at 37 °C for 20 min. The cells were washed with DMEM to remove the DCFH-DA and photoed using confocal microscope (488 nm/525 nm).

##### 2.2.3.6. Cell apoptosis

The percentage of apoptotic HCCLM3 cells was determined by Annexin V-FITC/PI detection kit based on the manufacturer’s protocol. In brief, HCCLM3 cells were cultured at a density of 1 × 10^5^ cells/mL in 12-well plates and incubated with Man-NIT and PEG-NIT micelles for 4 h. The cells were washed and collected. The cells were then transferred to a 1.5 mL tube after centrifugation at 1000 rpm for 5 min. FITC-conjugated Annexin V (5 mL) and propidium iodide (PI) (10 mL) were added to incubate with the cells for 20 min at room temperature. Finally, the stained cells were measured and analyzed by flow cytometry.

##### 2.2.3.7. Calcein AM-PI staining

HCCLM3 cells were cultured at a density of 1 × 10^4^ cells/mL in 24-well plates and incubated with Man-NIT and PEG-NIT micelles for 4 h. Then the cells were washed with PBS and 250 μL Calcein AM/PI solution was added for incubation at 37 °C for 30 min. The cells were washed with PBS and observed using fluorescence microscope (Calcein AM was green fluorescence, Ex/Em = 494/517 nm; PI is red fluorescence, Ex/Em = 535/617 nm).

#### 2.2.4. Animals experiment

##### 2.2.4.1. In vivo imaging

DiR were encapsulated with nanomicelles to prepare the Man-NIT@DiR, PEG-NIT@DiR in NaCl (1 mg/mL). BALB/c nude mice were divided into three groups and injected 100 μL DiR, Man-NIT@DiR, and PEG-NIT@DiR by tail vein. The nanomicelles distribution in nude mice were photographed by Carestream in vivo FX Professional Imaging System equipped with an excitation-pass filter at 760 nm and emission-pass filter at 790 nm. The protocol of exposure: X-ray exposure time was 30 s. The data were evaluated and statistically analyzed using the Carestream imaging system.

##### 2.2.4.2. Establishment of subcutaneous tumor model in nude mice

Twenty-four male BALB/c nude mice were conventionally fed in SPF sterile environment for one week. HCCLM3 cells (1 × 10^6^ cells/mL) in logarithmic growth phase were taken and injected subcutaneously into the back of nude mice with insulin needle. After building the model for 5 days, the model mice were divided into model, Man-NIT, and PEG-NIT group. The groups were administered by NaCl, Man-NIT and PEG-NIT (10 mg/mL, 20 mg/kg) through the tail vein daily, respectively. The treatment period was 21 days, and then the tumorigenesis of nude mice was observed daily. Another 6 unmodeled BALB/c nude mice were used as control.

##### 2.2.4.3. Tumor volume

The survival status of mice was observed daily, and the body weight of mice in each group was measured every 3 days. After 2 weeks of treatment, the growth of tumor in the three groups of nude mice was observed and the long diameter (a) and short diameter (b) of the tumor were measured. The calculation formula of tumor volume is V = a × b^2^/2.

##### 2.2.4.4. HE staining

After a series of processes including embedding, sectioning, baking, dewaxing, and hydration, the tumor tissues of nude mice were stained with hematoxylin solution and then stained with eosin. After dehydration, the tissue was mounted by neutral balsam and observed under an upright microscope.

##### 2.2.4.5. Expression of Ki67 protein detection by immunohistochemistry

The expression of Ki67 protein in tumor tissues of nude mice was detected using immunohistochemistry, and the operation was performed according to the manufacturer’s protocols. The tumor sections were treated with dewaxing, rehydration, antigen repair and blocking. The sections were incubated with the first antibody Ki67 (1:100) 4 °C overnight. The next day, the sections were incubated with secondary antibodies for 1 h. After the sections were stained with diaminobenzidine and photoed using fluorescence microscope.

## 3. Results

### 3.1. Synthesis of Man-NIT and PEG-NIT

**Figure 1.**
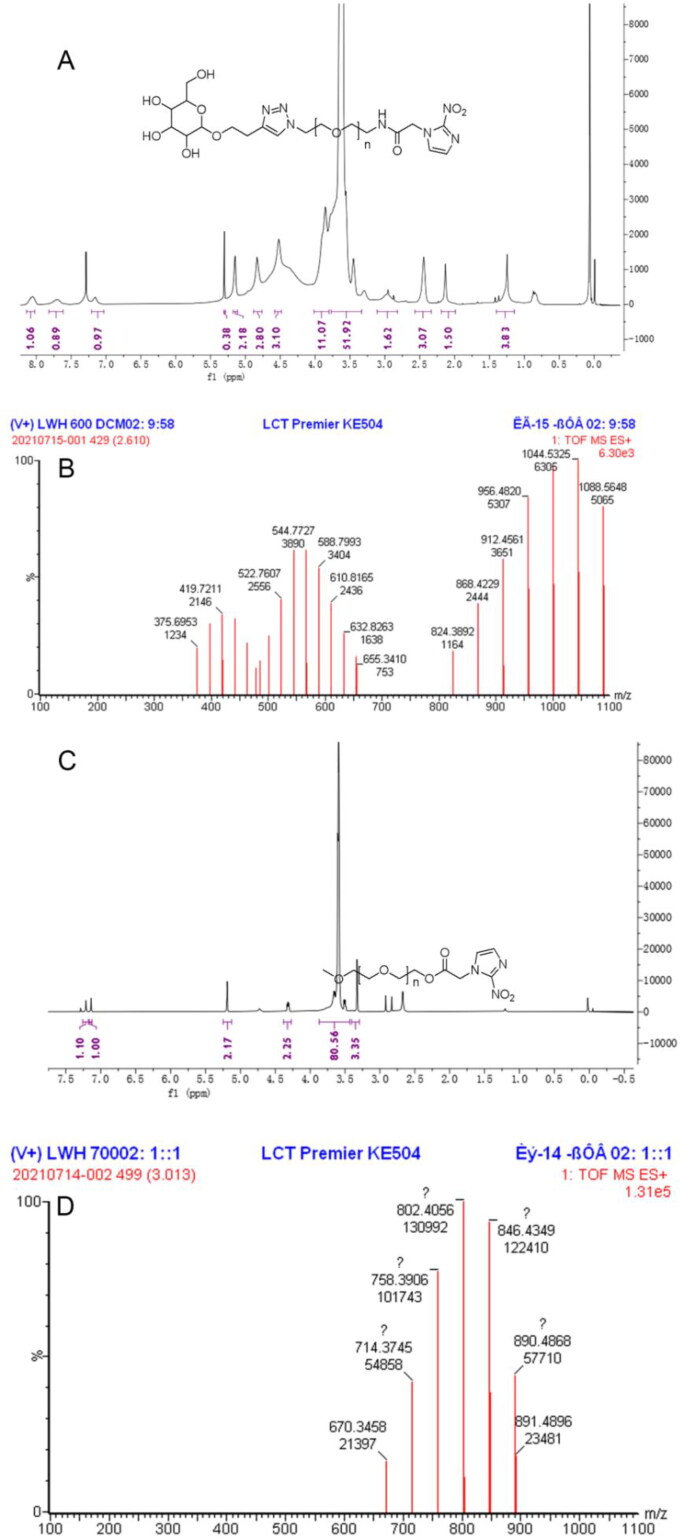
Synthesis of Man-NIT and PEG-NIT compounds. (A) ^1^H NMR spectrum of Man-NIT; (B) HRMS of Man-NIT; (C) ^1^H NMR spectrum of PEG-NIT; (D) HRMS of PEG-NIT.

The pure product Man-NIT (400 mg, 65.1%): ^1^H NMR (400 MHz, Chloroform-d) δ 8.14 − 8.02 (m, 1H), 7.70 (s, 1H), 7.21 − 7.03 (m, 1H), 5.30 (s, 1H), 5.15 (s, 2H), 4.83 (s, 3H), 4.52 (s, 3H), 4.02 − 3.80 (m, 11H), 3.77 − 3.33 (m, 52H), 3.11 − 2.82 (m, 2H), 2.44 (t, J = 6.5 Hz, 3H), 2.13 (s, 2H), 1.40 − 1.14 (m, 4H). HRMS (ESI) Calcd for C_19 + 2n)_H_(29 + 4n)_N_7_O_(10+n)_ [M + H]+, 956.5, 1044.5, 1088.6 et al.; found 956.5, 1044.5, 1088.6 (Figure 1(A,B)). The pure product PEG-NIT (480 mg, 79.7%): ^1^H NMR (400 MHz, Chloroform-d) δ 7.21 (s, 1H), 7.14 (s, 1H), 5.19 (s, 2H), 4.38 − 4.27 (m, 2H), 3.87 − 3.43 (m, 81H), 3.33 (s, 3H), HRMS (ESI) Calcd for C_(10 + 2n)_H_(15 + 4n)_N_3_O_(6+n)_ [M + H]^+^ 802.4, 846.4, 890.5 et al.; found 802.4, 846.4, 890.5 et al. (Figure 1(C,D)).

### 3.2. Characterization of nanomicelles

**Figure 2.**
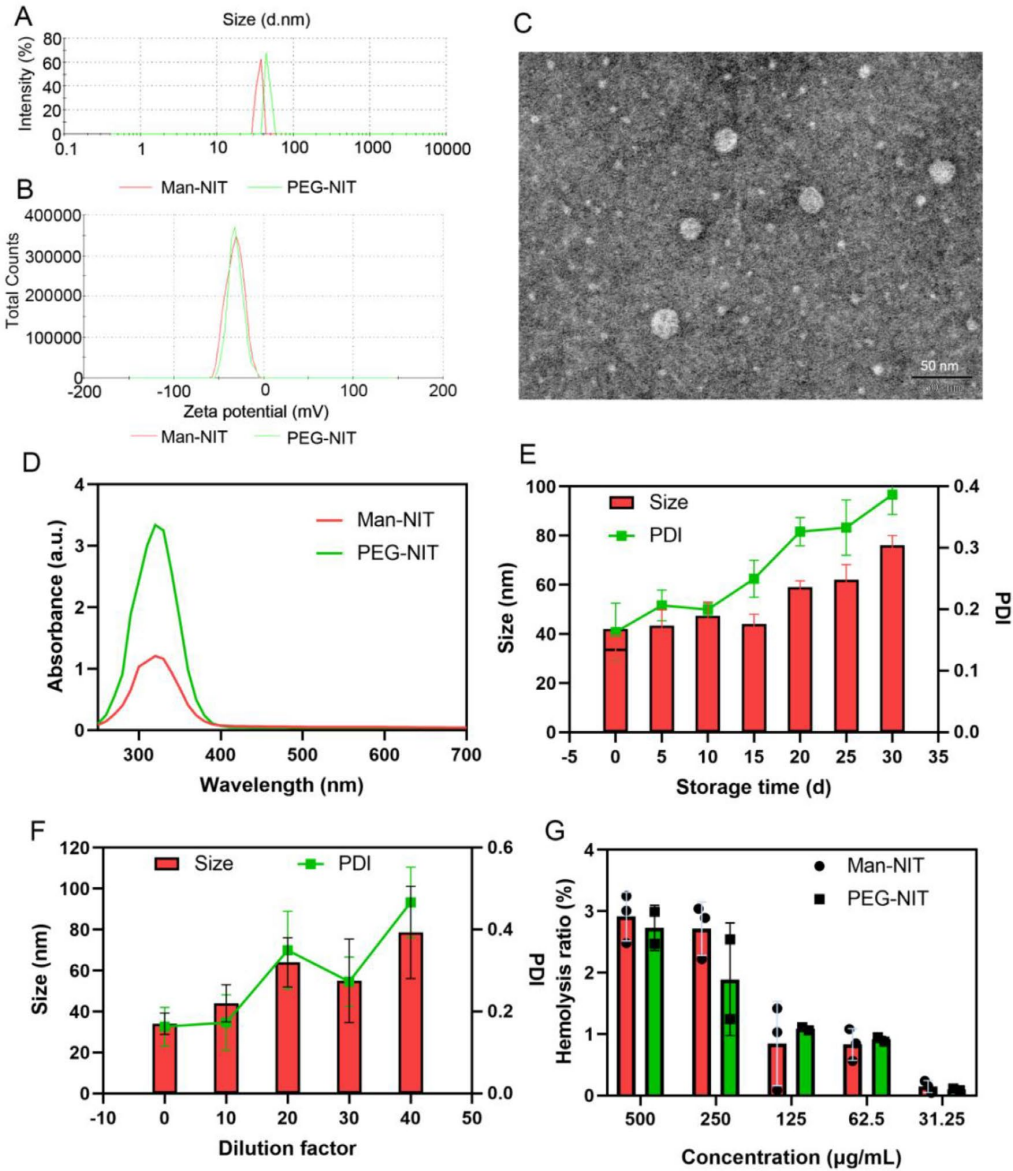
Characterization of nanomicelles. (A) Particle size distribution; (B) ζ-potential; (C) TEM image of Man-NIT micelles; (D) Ultraviolet absorbance (a.u.); (E) Stability of nanomicelles during 30 days. (F) Stability of Man-NIT micelles after dilution. (G) Hemolysis ratio (%).

The average particle size of Man-NIT and PEG-NIT were 34.7 ± 1.20 nm and 46.2 ± 2.56 nm. PDI of Man-NIT and PEG-NIT were 0.184 ± 0.05, 0.193 ± 0.06, respectively. The particle size distribution and ζ-potential were shown in Figure 2(A,B). Nanomicelles were spherical in the TEM image (Figure 2(C)). The characteristic of these particles exhibit no difference between Man-NIT and PEG-NIT (p > 0.05). Full wavelength scan displayed that the maximum UV absorption of Man-NIT and PEG-NIT was 320 nm (Figure 2(D)). Man-NIT micelles were stored at 4 °C for 30 days and the particle size and PDI of nanomicelles were detected at 0, 5, 10, 15, 20, 25 and 30 day, respectively. It showed that there was no much change in size and PDI of Man-NIT and PEG-NIT before 20 days. However, the size and PDI slightly increased after the 20th day (Figure 2(E)). The measured particle size and PDI results of Man-NIT micelles after dilution by 10, 20, 30, and 40 times were shown in Figure 2(F). With the increase of dilution times, the particle size and PDI of nanomicelles increased slightly, which was most obvious when the dilution was up to 500 times. Hemolysis ratio of Man-NIT and PEG-NIT micelles were less than 5%, as shown in Figure 2(G). There was no significant difference between Man-NIT and PEG-NIT (p > 0.05). It indicated that the nanomicelles had no hemolysis and were safe for intravenous administration.

### 3.3. Effect of nanomicelles on HCCLM3 cells

**Figure 3.**
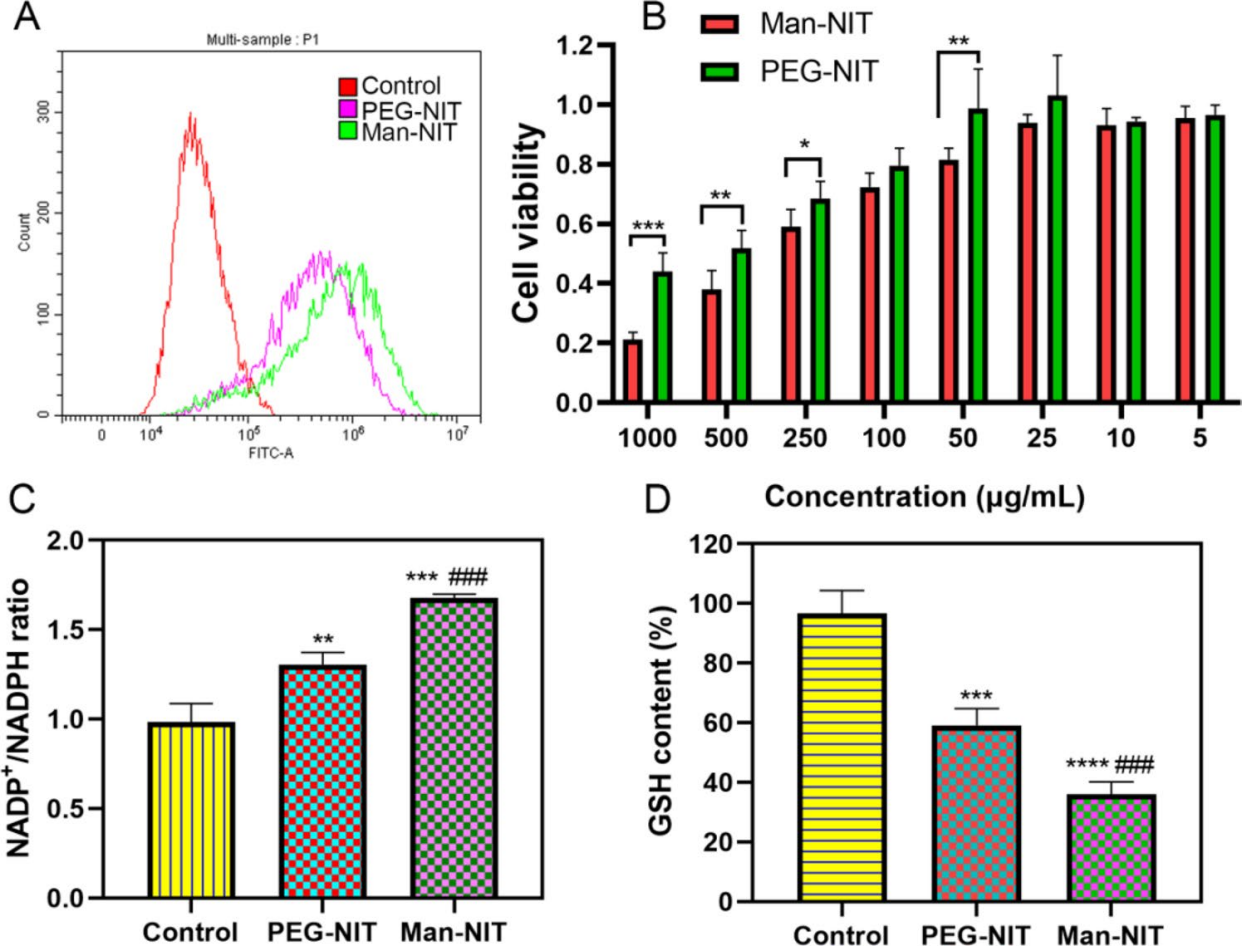
Effect of nanomicelles on HCCLM3 cells. (A) Cellular uptake of Dio-labelled nanomicelles using flow cytomertry; (B) Cell viability; (C) NADP^+^/NADPH ratio, compared with control, **p < 0.01, ***p < 0.001, compared with PEG-NIT, ^###^p < 0.001; (D) GSH content. compared with control, ***p < 0.001, ^****^p < 0.0001, compared with PEG-NIT, ^###^p < 0.001.

**Figure 4.**
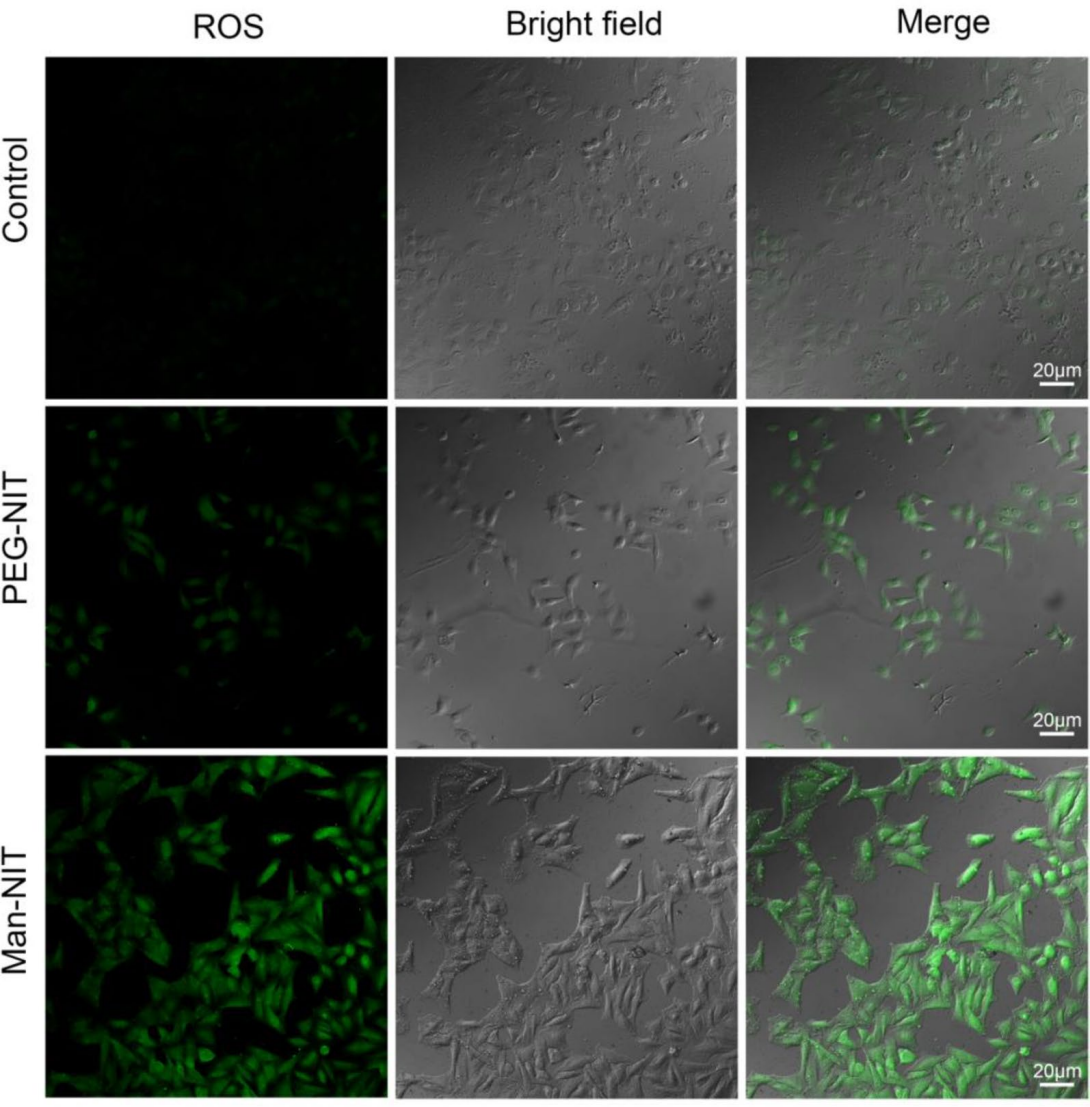
ROS fluorescence images in HCCLM3 cells after giving nanomicelles. Scale bar: 20 μm.

Flow cytometry showed that Man-NIT micelles were more cellular uptake than that of PEG-NIT micelles (Figure 3(A)). Cell viability was analyzed using MTT assay. It was found that the cell viability decreased gradually with the increased concentration of nanomicelles. The cell viability of Man-NIT micelles was only 0.21 ± 0.024 at the concentration of 1 mg/mL, while PEG-NIT was 0.44 ± 0.062. When the concentration was reduced to 50 μg/mL, there was no obvious inhibitory effect on cell proliferation (Figure 3(B)). After HCCLM3 cells being incubated with nanomicelles, the NADPH content in Man-NIT group obviously decreased, which presented as NADP^+^/NADPH ratio. Compared with control group, the GSH contents of Man-NIT and PEG-NIT groups were decreased, and there was a significant difference, ***p < 0.001, ^****^p < *0*.0001 (Figure 3(C,D)). Compared with the control group, ROS increased more in both Man-NIT and PEG-NIT groups, especially the Man-NIT group (Figure 4). The above consumption of NADPH, reduction of GSH content and increase of ROS all cause the imbalance of redox in tumor cells, and eventually induce the death of cells.

### 3.4. Cell apoptosis and calcein AM-PI staining

**Figure 5.**
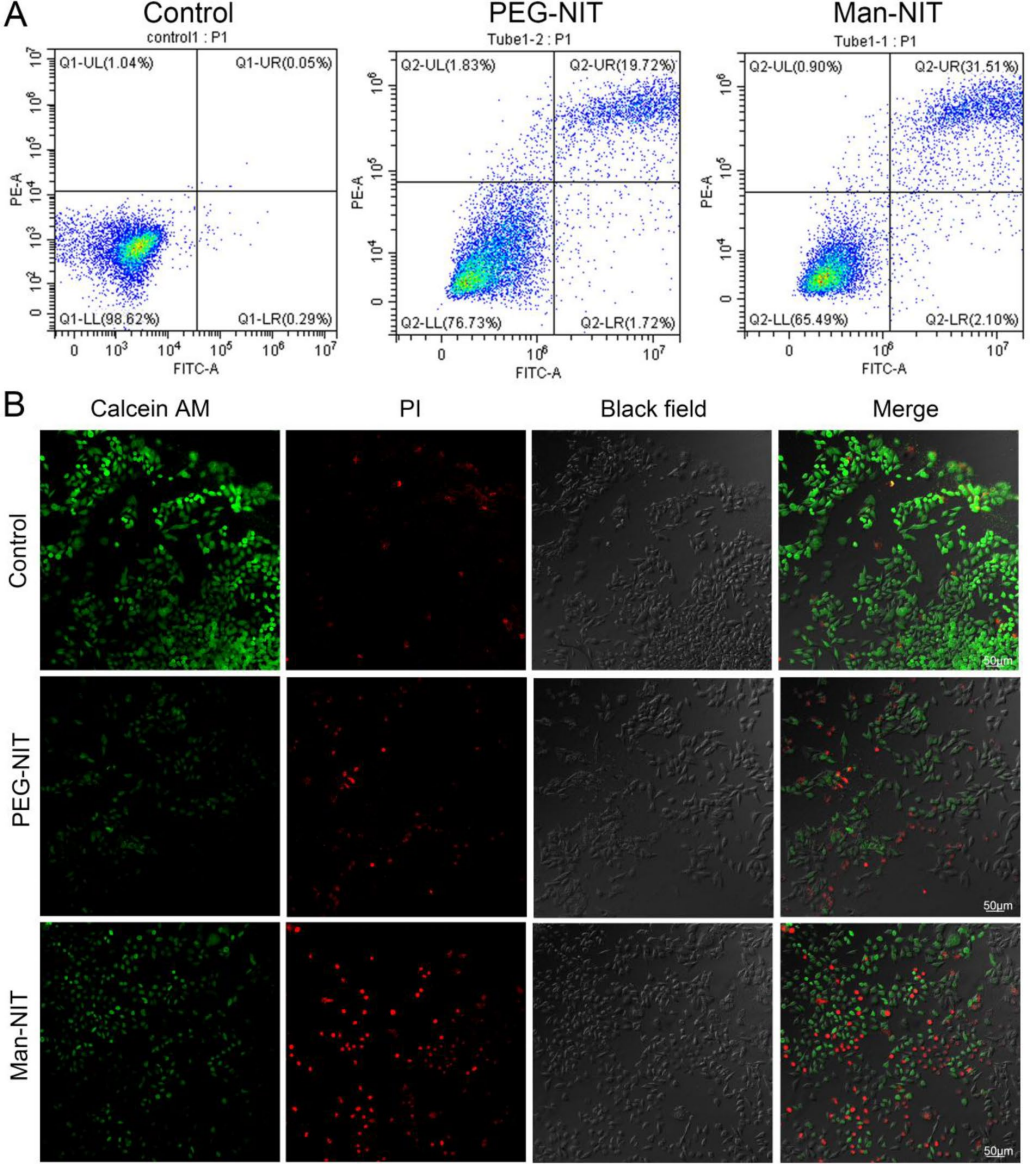
Cell apoptosis and Calcein AM-PI staining. (A) Flow cytometiric analysis apoptosis; (B) Fluorescence images of Calcein AM-PI staining, Scale bar: 50 μm.

Flow cytometry showed that the apoptosis rate of HCCLM3 cells either treated with PEG-NIT or Man-NIT was 19.72% and 31.51%, as shown in Figure 5(A). In addition, HCCLM3 cells cultured with different nanomicelles were stained by Calcein AM-PI. Viable cells were stained with Calcein-AM (green), and dead/late apoptotic cells were stained with propidium iodide (PI) (red). The control group mainly showed green fluorescence, but PEG-NIT and Man-NIT groups showed red fluorescence imaging. Among the apoptotic cells in Man-NIT group were obvious (Figure 5(B)).

### 3.5. Targeting of nanomicelles

**Figure 6.**
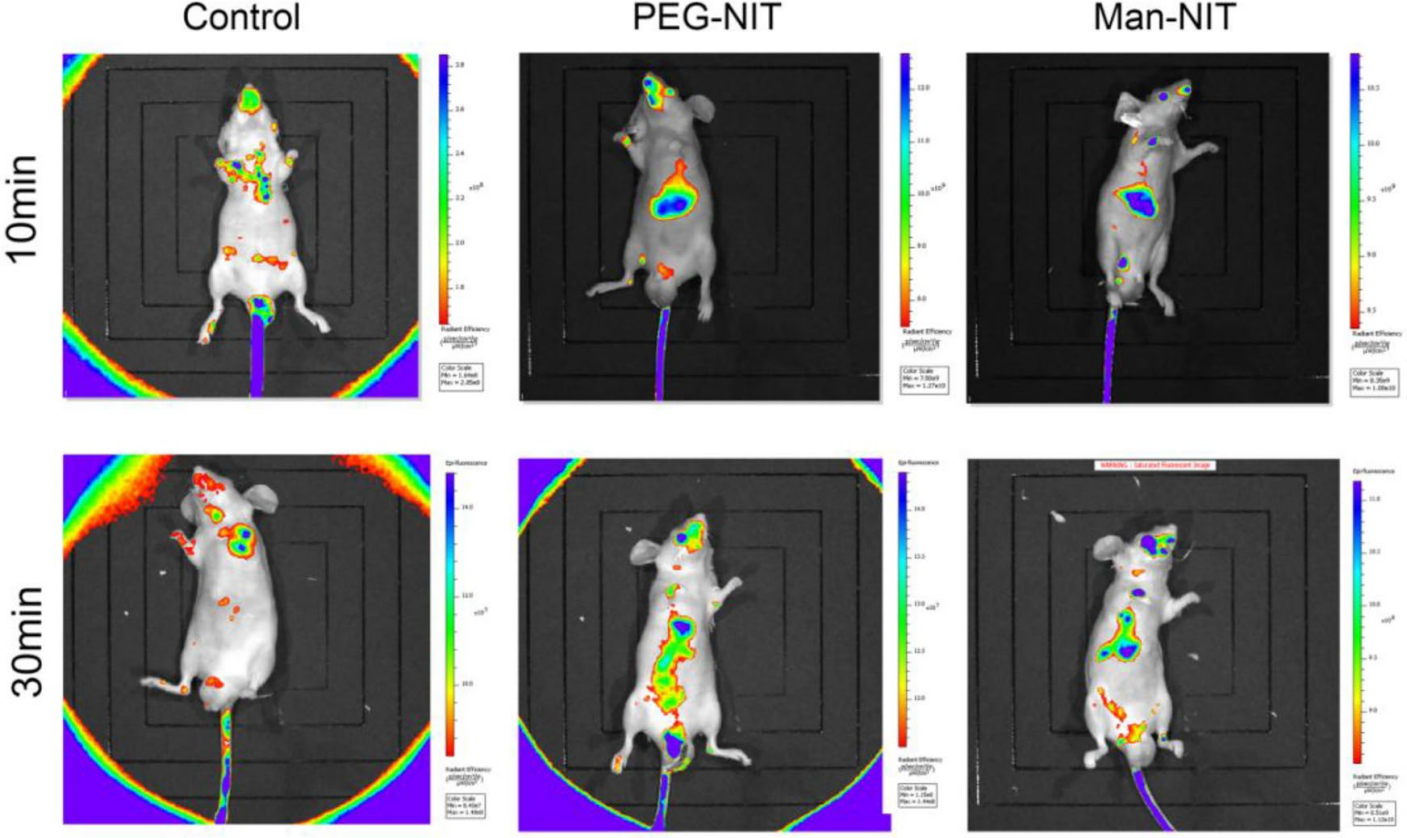
Targeting distribution of various DiR-labeled of nanomicelles in nude mice.

The nanomicelle distribution was observed in Figure 6. Free DiR was distributed irregularly in the control mice, but PEG-NIT@DiR and Man-NIT@DiR showed the distribution of nanomicelles was mainly in the liver of mice. It can be used to deliver nanomicelles to the liver for targeted HCC therapy.

### 3.6. Assessment of tumor size and pathological characteristics

**Figure 7.**
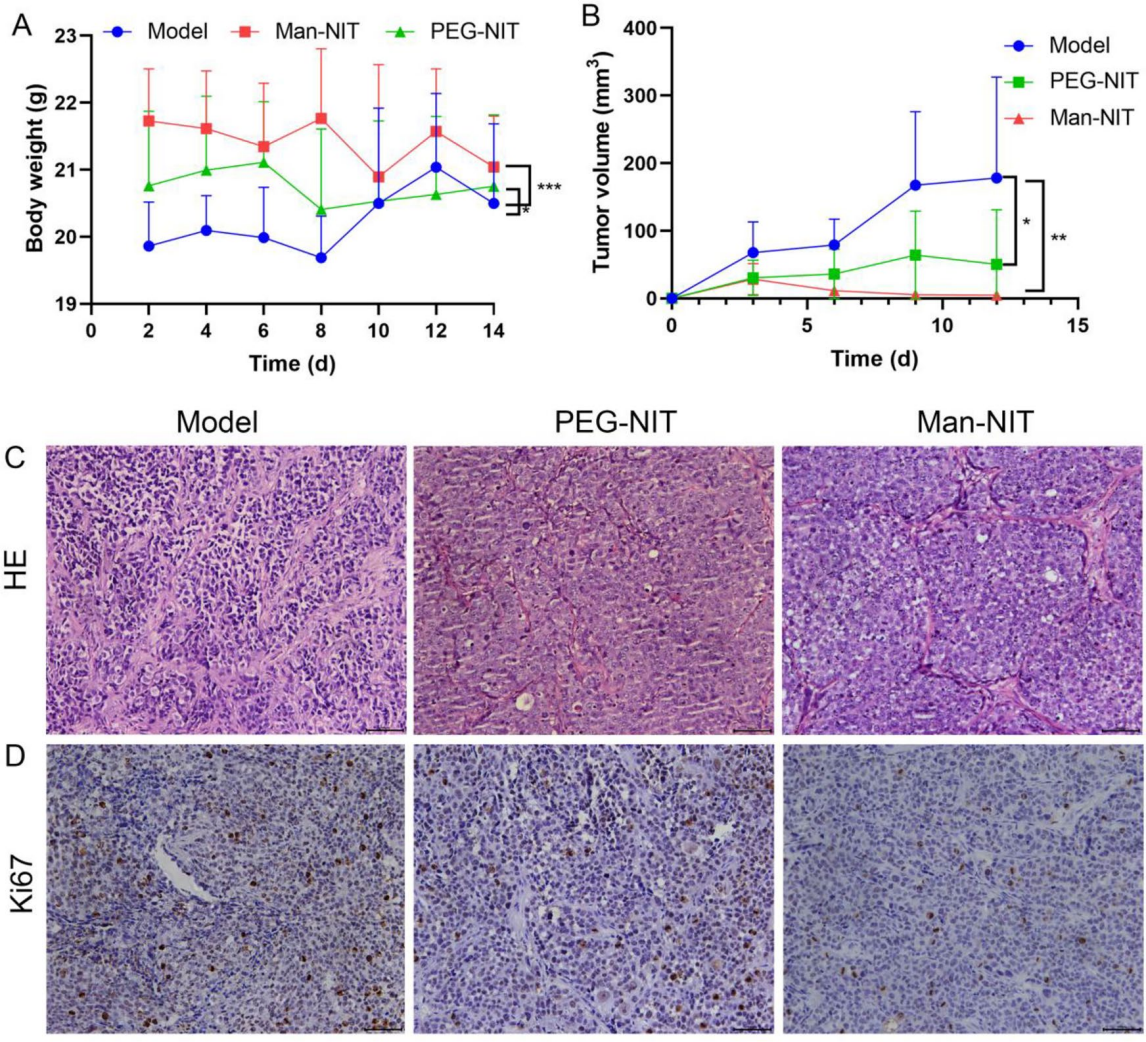
Tumor size and pathological characteristics after nanomicelles treatment for nude mice. (A) Body weight (g); (B) Tumor volume (mm^3^); (C) HE staining of tumor tissues (400×); (D) Immunohistochemical detection of Ki67 protein in tumor tissues, scale bars: 400×.

After 14-day of nanomicelles treatment in nude mice, the body weight of mice in each group was shown in Figure 7(A). The body weight of mice in the model group was significantly lower than that of Man-NIT and PEG-NIT groups. Tumor volume in different groups were shown in Figure 7(B). It can be seen that nanomicelles could inhibit tumor growth after 14-day therapy. In brief, with the extension of time, the tumors in the model group gradually increased, but in Man-NIT group they significantly decreased. After HE staining of tumor tissues in nude mice, the nuclei were stained dark blue and the cytoplasm was light red. The pathological characteristics of tumor tissues showed that apoptotic cells, nuclear pyknosis, and nuclear fragmentation occurred in the nanomicelle groups, especially the Man-NIT group. However, the nuclei of the model group was complete and clear, and stained uniformly blue (Figure 7(C)). Ki67 is nuclear positive, and the brown dot in image shows Ki67 protein expression. High Ki67 expression means higher malignancy. The levels of Ki67 were much higher in model group than that in PEG-NIT and Man-NIT groups (Figure 7(D)). It indicates that namomicelles have a tumor inhibition effect, especially Man-NIT.

### 3.7. Effects of nanomicelles on kidney and liver

**Figure 8.**
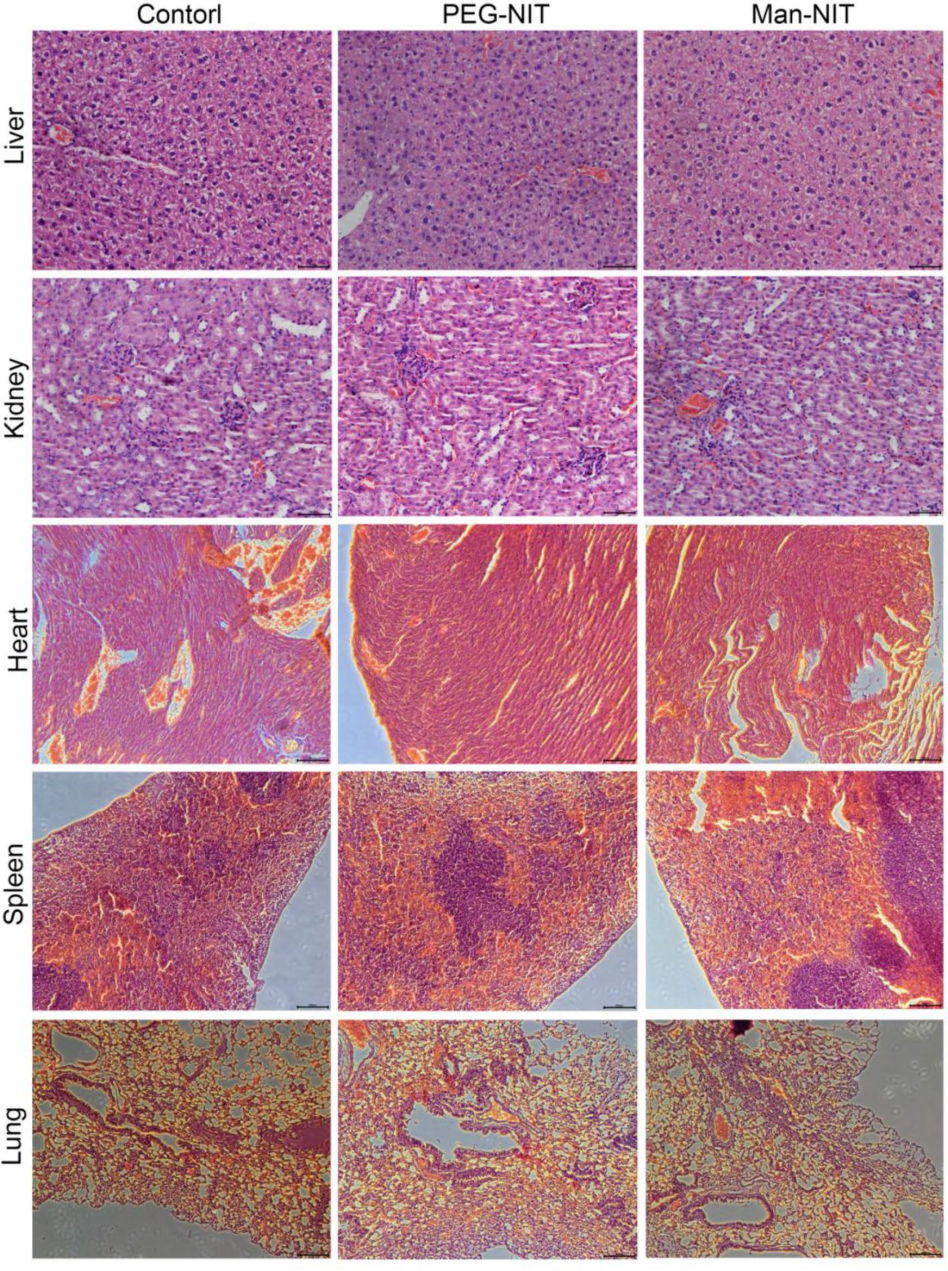
HE staining of liver, kidney, heart, spleen and lung tissue in nude mice (400×).

HE staining showed that the lung cells of mice were evenly arranged with uniform density and no pathological cells in Figure 8(A). The renal cells of mice were evenly arranged, and clear glomeruli and tubules could be seen in Figure 8(B). It indicates that nanomicelles are relatively safe and harmless to major metabolic organs.

## 4. Discussion

In this experiment, the nanodrug Man-NIT micelles were firstly designed and synthesized. Man-NIT micelles not only exhibited nano-sized particle size and excellent stability, but also no toxicity and hemolysis. Mannose as a transport substrate of GLUT1, which could be used to modify the nanomicelles. We found that Man-NIT micelles could be taken more in by HCCLM3 cells after modification than that of PEG-NIT without mannose modification. The reason is that compared with normal cells, the tumor cells need significantly enhanced glucose uptake ability and metabolic activity to satisfy the purpose of rapid proliferation, namely Warburg effect (Poff et al., 2019). In order to adapt to Warburg effect, tumor cells will overexpress multiple transporters, such as GLUUT1, solute carrier family 2A member 1 (SLC2A1) and so on. When glucose is sufficient, tumor cells mainly rely on GLUT1 for uptake, because this method is more economical for tumor cells (Zhao et al., 2018).

Man-NIT not only has the excellent performance of nanocarrier, but also exhibits the capability of antitumor. With the increase of nanomicelle concentration, Man-NIT can effectively inhibit tumor growth. The nitroimidazole of Man-NIT will deplete NADPH under the action of nitrogenase. Our results showed that the content of NADPH in Man-NIT group was obviously decreased, which was consistent with the literature (Guo et al., 2020). It also reports that the increased production of NADPH and GSH can counteract oxidative stress and promote tumor cell survival and growth in mice (Tong et al., 2021). GSH can scavenge free radicals and peroxides, hence decline of GSH can lead to excessive ROS generation and cell apoptosis (Armstrong & Jones, 2002). GSSG is reduced to GSH by hydrogen supplied from NADPH under the action of glutathione reductase (GR). Therefore, NADPH plays an important role in maintaining the content of GSH in cells as a coenzyme of GR (Mejía et al., 2018; Moreno-Sanchez et al., 2018). So Man-NIT attenuates oxidative stress antioxidants, such as GSH, and inhibits tumor proliferation. When HCCLM3 cells were incubated with nanomicelles, NADPH depletion in cells would inevitably affect GSH synthesis, resulting in the decline of GSH content. Compared with control group, the GSH content of Man-NIT and PEG-NIT group decreased, which due to both nanomicelles containing nitroimidazole structure. Because of the blocked synthesis of GSH, the ability of counteracting oxidative stress and scavenging ROS in tumor cells will also be weakened. We used the fluorescent probe DCFH-DA to detect ROS and found ROS increased after HCCLM3 cells incubated with Man-NIT. If the high concentration of ROS in tumor cells cannot be timely reduced by GSH, it will lead to the imbalance of redox in cells and even tumor cell death. Man-NIT can promote the productions of ROS via NADPH depletion and decreasing GSH, subsequently aggravating lipid peroxidation and triggering cell death in HCC.

Imaging in vivo showed that Man-NIT could target the liver and mainly accumulated in the liver of mice, which was closely related to the high expression of GLUT1 on HCC cells (Liu et al., 2019). In addition, GLUT1 on tumor cells showed a higher transport rate than that on normal cells (Amann & Hellerbrand, 2009; Amann et al., 2009). Compared with receptor-mediated cellular uptake, transporter mediated cellular uptake has a faster transport rate and higher specificity. Therefore, nanomicelles modified by mannose, a GLUT1 transport substrate, are easily to target the liver. These superior features of Man-NIT can be used to deliver nanomicelles to the liver for GLUT1-targeted HCC therapy. In order to further investigate the in vivo therapeutic effect of nanomicelles on HCC, nude mice were treated with nanomicelles for 14 days. After 14-day therapy, it was found that the tumors in the model group gradually increased, but in the Man-NIT treated group, the tumor size was significantly decreased. Similarly, HE staining showed that the apoptotic cells appeared in tumor tissues after nanomicelles treatment. Ki67 immunohistochemistry showed that Ki67 protein was highly expressed in model group of nude mice, which meant tumor tissues in the model having a fast proliferation. However, the above pathological characteristics were significantly improved after Man-NIT administration. It indicates that namomicelles have a tumor inhibition effect especially Man-NIT. Moreover, we dissected other major metabolic organs of nude mice, such as liver and kidney, and found that nanomicelles had no toxic and side effects on these metabolic organs during treatment. As a new self-assembled nanomicelles system, Man-NIT may become a potential chemotherapeutic drug for HCC therapy and a delivery platform for related nanodrugs.

## 5. Conclusion

A structurally novel self-assembled nanomicelles, namely Man-NIT, was designed and synthesized. The outer shell was mannose, which could target GLUT1 on the tumor cells. While the inner core nitroimidazole moiety of Man-NIT could react with NADPH, resulting in decrease of GSH and enhancement of ROS to disturb the balance of redox in tumor cells, and finally caused the tumor cells death. It indicates that Man-NIT micelles have the abilities of tumor inhibition, which may be a potential nanodrug of chemotherapeutics for HCC therapy.
